# Genetic Variants of the *IL‐23*/*IL‐17* Axis and Its Association With Periodontal Disease: A Systematic Review

**DOI:** 10.1002/iid3.70147

**Published:** 2025-01-31

**Authors:** Ruth Rodríguez‐Montaño, Mario Alberto Alarcón‐Sánchez, Sarah Monserrat Lomelí‐Martínez, Cristina Hermila Martínez‐Bugarin, Artak Heboyan

**Affiliations:** ^1^ Department of Health and Illness as an Individual and Collective Process University Center of Tlajomulco, University of Guadalajara (CUTLAJO‐UdeG) Tlajomulco de Zuñiga Mexico; ^2^ Institute of Research in Dentistry, Department of Integral Dental Clinics University Center of Health Sciences, University of Guadalajara Guadalajara Mexico; ^3^ Molecular Biology Department, University Center of Health Sciences University of Guadalajara Guadalajara Mexico; ^4^ Department of Medical and Life Sciences La Ciénega University Center, University of Guadalajara Jalisco Mexico; ^5^ Department of Research Analytics, Saveetha Dental College and Hospitals, Saveetha Institute of Medical and Technical Sciences Saveetha University Chennai India; ^6^ Department of Prosthodontics, Faculty of Stomatology Yerevan State Medical University after Mkhitar Heratsi Yerevan Armenia; ^7^ Department of Prosthodontics, School of Dentistry Tehran University of Medical Sciences North Karegar St Tehran Iran

**Keywords:** cytokines, genes, IL‐17, IL‐23, peri‐implantitis, periodontitis

## Abstract

**Background:**

The objective of this systematic review was to identify genetic variants of the *IL‐23*, *IL‐17*, *IL‐23R* and *IL‐17R* genes and isoforms and its possible association with increased development of periodontitis and peri‐implantitis.

**Methods:**

A systematic review was prepared according to the guidelines, registered in the OSF database with the registration number: 10.17605/OSF. IO/X95ZC. The electronic search was performed in four databases: PubMed, Scopus, Web of Science, and Google Scholar from 1984 until March 15th, 2024. The JBI Critical Appraisal Checklist for Case‐Control Studies was used to assess the quality of included studies.

**Results:**

Eighteen papers with a case‐control design were those that ultimately met the eligibility criteria. A total of 3904 individuals (2315 with periodontitis and 90 with peri‐implantitis), and 1589 healthy subjects) were studied. The age range of the study population was 14–70 years, with a mean age ± (SD) of 40.43 ± 6.33 years. A total of 28 genetic variants corresponding to the *IL‐17A* (rs 2275913, rs 3819024, rs 10484879) *IL‐17F* (rs 763780), *IL‐17R* (rs 879576) and *IL‐23R* (rs 11209026) genes were analyzed in this study. Six (33.3%) studies found an association between the *IL‐17A* 197 G/A (rs 2275913) genetic variant and peri‐implantitis and periodontitis. One study (5.5%) found an association between the *IL‐17A* rs10484879 variant and peri‐implantitis and periodontitis.

**Conclusion:**

Six polymorphisms were evaluated, highlighting rs 2275913 of the cytokine IL‐17A in patients with periodontitis or peri‐implantitis. Only 50% of studies found an association despite having a small sample. This suggests that other factors such as the degree of disease, systemic diseases and ethnic groups studied may play a role.

**Abbreviations**
COVID‐19: Coronavirus disease 2019
GM‐CSF: granulocyte‐macrophage colony‐stimulating factor
IFN‐γ: interferon gamma
IL‐17A: interleukin 17A
IL‐17B: interleukin 17B
IL‐17C: interleukin 17C
IL‐17D: interleukin 17D
IL‐17E: interleukin 17E
IL‐17F: interleukin 17F
IL‐17R: interleukin 17 receptor
IL‐17RA: interleukin 17 receptor A
IL‐17RC: interleukin 17 receptor C
IL‐21: interleukin 21
IL‐22: interleukin 22
IL‐23: interleukin 23
IL‐23R: interleukin 23 receptor
IL‐25: interleukin 25
IL‐6: interleukin 6
iNKT: invariant natural killer T cells
JAK2: Janus kinase 2
LTi: lymphoid tissue inducer cells
MMPs: matrix metalloproteases
NK: natural killer cells
PCR: polymerase chain reaction
RANKL: receptor activator for nuclear factor kappa B ligand
RFLP: restriction fragment length polymorphism
RORγT: RAR‐related orphan receptor gamma
STAT3: signal transducer and activator of transcription 3
TGF‐β: transforming growth factor beta
Th17 cells: T helper 17 cells
TNF‐α: tumor necrosis factor alpha
Tγδ: gamma delta T cells
VEGF: vascular endothelial growth factor

## 1 Introduction

The progressive degradation of the supporting structure of the teeth is a hallmark of periodontitis, caused mainly by microbial dysbiosis [1]. On the other hand, peri‐implantitis is a condition of the tissues surrounding dental implants. It presents inflammation of the peri‐implant mucosa and gradual loss of supporting bone [2].

Although these two illnesses affect the periodontal tissue, they share some traits, the main one is dysbiosis which results in gingival inflammation. Periodontitis and peri‐implantitis can be caused by a variety of risk factors, including smoking, genetic predispositions, poor patient selection, insufficient periodontal therapy, and failure to diagnose and treat peri‐implant mucositis, among other aspects [3, 4].

On the other hand, the immune system is highly involved both in maintaining health and in the imbalance. In this sense, it is known that the IL‐23/IL‐17 axis is highly present in periodontal tissues. The cytokines IL‐23 and IL‐17 and their receptors are usually overexpressed in situations where an extracellular pathogen like bacteria or fungus. Th17 cells are the cornerstone of this axis, cells responsible for coordinating the elimination of pathogens that damage periodontal tissues [5, 6].

Over the past decades, a subset of CD4^+^ T cells known as Th17 has been identified, distinguished primarily by their production of IL‐17 [5, 6, 7].

Th17 cells play a fundamental role in the response against extracellular growing bacteria and fungi [8]. Th17 cell activity is regulated by cytokines such as IL‐23, TGF‐β, and IL‐6. In this sense, IL‐23 plays a key role in proliferation and differentiation [5].

IL‐23 is generated by many cells, primarily dendritic cells, which detect infections and drive these cells to release various pro‐inflammatory cytokines, including IL‐23. IL‐23 is a heterodimeric cytokine consisting of two subunits linked by a disulfide bond: the soluble p40 subunit and the p19 component, which forms a tetra‐helical bundle [9, 10]. When IL‐23 binds to its receptor on Th17 cells, it activates the RORγt^+^ transcription factor, leading to the overexpression of IL‐23R on the cell membrane. This creates a positive feedback loop that supports the maintenance and clonal expansion of Th17 cells [11]. Once activated, Th17 cells produce the cytokine IL‐17 such as RANKL, GM‐CSF, TNF‐α, IFN‐γ, IL‐21, and IL‐22 [12].

There are six known IL‐17 molecules named A to F. IL‐17A is considered the main member of the IL‐17 family and, therefore, the most investigated [13, 14].

Importantly, the cytokines IL‐17A and IL‐17F are secreted by immune cells such as Th17, LTi, NK, iNKT cells, mast cells, neutrophils and γδ T cells [15].

By other part, IL‐17A and IL‐17F can assemble into homodimers (IL‐17A/A or IL‐17F/F) or heterodimers (IL‐17A/F) [16, 17].

The 6 isoforms of IL‐17 are recognized by five receptors (A–E). The IL‐17RA and IL‐17RC receptors form a heterodimer that has the ability to recognize IL‐17A as well as the heterodimer formed by IL‐17A/IL‐17F. The IL‐17RA receptor is ubiquitously expressed and IL‐17RC is expressed in epithelial cells, fibroblasts, chondrocytes and adipocytes [13, 18, 19].

Nonimmune cells such as fibroblasts can be activated by IL‐17A and IL‐17F. In turn, fibroblasts induce different pro‐inflammatory mediators such as cytokines, chemokines, MMP, VEGF, RANKL and antimicrobial peptides [20].

Currently, several studies have evaluated the levels of IL‐23 and IL‐17, including their receptors, in patients with periodontitis in different biological samples such as gingival tissue [21, 22, 23], serum [24, 25], plasma [26, 27, 28], saliva [29, 30] and gingival crevicular fluid [23, 25, 31, 32, 33]. Although most have concluded that IL‐23 and IL‐17A are elevated in patients with periodontitis unlike healthy subjects, some authors have disagreed or have not observed significant differences [34, 35, 36, 37, 38, 39, 40]. Regarding this, some genetic variants may cause the deregulation in the expression of cytokines of the IL‐23/IL‐17 axis. The objective of this systematic review was to identify genetic variants of the *IL‐23*, *IL‐17*, *IL‐23R* and *IL‐17R* genes and isoforms and its possible association with increased development of periodontitis and peri‐implantitis.

## 2 Materials and Methods

This systematic review was prepared and reported following the Preferred Reporting Items for Systematic Reviews and Meta‐Analyses (PRISMA) [41] guidelines and was registered in the Open Science Framework (OSF) database with the registration number: 10.17605/OSF. IO/X95ZC.

### 2.1 Researcher Question

Is there any relationship between to variants of the *IL‐23*, *IL‐17*, *IL‐23R* and *IL‐17R* genes and isoforms and the risk of developing periodontitis and/or peri‐implantitis?

### 2.2 PECO Outline

- Population: Healthy subjects without systemic disease.
- Exposure: Genotypes and allelic distribution of the different genetic variants of genes *IL‐23* and *IL‐17* and its receptors *IL‐23R* and *IL‐17R* between the exposure and control groups. In addition, to emphasize its possible association with clinical parameters.
- Control: Subjects without periodontitis and/or peri‐implantitis.
- Outcomes: Subjects with periodontitis and/or peri‐implantitis.

### 2.3 Eligibility Criteria

Clinical case‐control studies were included, as well as genetic association studies analyzing any genetic variant of the *IL‐23*, *IL‐17*, *IL‐23R* and *IL‐17R* genes and isoforms in subjects with periodontitis and/or peri‐implantitis. Studies without control groups were excluded, as well as book chapters, thesis, abstracts, letters to the editor, short communications, mini‐reviews, narrative reviews, scoping reviews, comprehensive reviews, meta‐analyses, and conference posters.

### 2.4 Literature Search and Study Selection

An electronic search without language restriction was conducted in four databases: PubMed [2011–2022], Scopus [2012–2022], Web of Science [2013–2015], and Google Scholar [1984–2024] until March 15th, 2024. Depending on the database consulted, keywords identified from the following MeSH (Medical Subject Headings) terms were used: “Polymorphism,” “Polymorphism Single Nucleotide,” “Interleukin 23,” “Interleukin 17,” “Interleukin receptor 23,” “Interleukin receptor 17,” ‘Periodontitis,’ and ‘Peri‐implantitis’ along with the use of Boolean operators ‘OR’ and ‘AND’. Some Journals related to the area (“*Oral Disease*,” “*International Journal of Periodontics & Restorative Dentistry*,” “*Periodontology 2000,*” “*Journal of Periodontal and Implant Science*,” “*Journal of Clinical Periodontology*,”, “*Journal of Periodontal Research*” y “*Journal of Periodontology*”), were also manually consulted, as well as additional searches were performed in the reference lists of all included studies to enrich the search strategy and ensure the reliability of the collected data. Table 1 shows the search strategy employed for all the databases.

**Table 1:** Electronic databases and search strategy.

Data base	Search strategy
**PubMed** (*n* = 41)	(((((((((“Polymorphism, Genetic”[Mesh Terms]) OR “Polymorphism, Single Nucleotide”[Mesh Terms]) OR “Genetic Variation”[Mesh Terms]) AND “Interleukin‐23”[Mesh Terms]) AND “Interleukin‐17”[Mesh Terms]) AND “IL23R protein, human” [Supplementary Concept]) AND “Receptors, Interleukin‐17”[Mesh Terms]) AND “Periodontitis”[Mesh Terms]) OR “Chronic Periodontitis”[Mesh Terms]) AND “Peri‐Implantitis”[Mesh Terms].
**Scopus** (*n* = 21)	TITLE‐ABS‐KEY (polymorphisms, AND genetic OR gene AND polymorphism AND il‐23 OR il‐17 AND periodontitis OR peri‐implantitis).
**Web of Science** (*n* = 2)	TS = (Single‐nucleotide polymorphism AND Interleukin 23 AND Interleukin 17 AND Periodontitis).
**Google Scholar** (*n* = 943)	(Polymorphisms, Genetic OR Gene Polymorphism OR Gene Polymorphisms OR Polymorphism, Gene OR Polymorphisms, Gene OR AND Interleukin 23 OR IL‐23 AND Interleukin 17 OR IL‐17 OR Interleukin 17A OR IL‐17A AND Periodontal Disease OR Periodontitis OR Chronic Periodontitis OR Aggressive Periodontitis OR Peri‐Implantitis).

The records obtained were imported into the EndNote V.9 program for subsequent analysis. In this way, two researchers (M.A.A.S. and R.R.M.) examined the titles and abstracts of the studies independently. Then, by reading the titles, duplicates were discarded and applying the eligibility criteria, a full‐text analysis of potentially eligible articles was performed. Disagreements were resolved by group discussion.

### 2.5 Data Extraction

A.H. and M.A.A.S. performed data extraction independently. Variables of interest data were extracted from the articles in tables prepared with Excel software (Microsoft 365). The data extracted from each article were the first author and year of publication, study design, country, gender, age, the number of cases, controls and total study population, periodontal status, genetic variants of the of the *IL‐23*, *IL‐17*, *IL‐23R* and *IL‐17R* genes and isoforms, type of sample, genotyping method and the main results obtained (Table 2).

**Table 2:** Main characteristics and outcomes of included studies.

Author's and Year	Study design	Population	Gender: F^e^/M^a^	Age (Mean/Range)	Cases/Controls	Total	Periodontal status	Gene/variant	Genotyping method	Outcome
Talib and Taha, 2024 [42]	Case‐controls	Iraqi	16/29	40.8	15/30	45	Peri‐implantitis	*IL‐17A*: 197 G/A (rs2275913)	PCR, sequencing	Associated
Alsherif et al. 2023 [43]	Case‐ controls	Lybian	50/50	25–65	50/50	100	Periodontitis	*IL‐17F*: +7488 C/T (rs763780)	PCR	Not associated
Malvandi et al. 2022 [44]	Case‐controls	Iranian	69/103	39.57	54/118	172	Periodontitis	*IL‐17A*: 197 G/A (rs2275913)	RFLP, PCR	Not associated
Mlachkova et al. 2021 [45]	Case‐controls	Bulgarian	33/17	46	40/10	50	Periodontitis	*IL‐17F*: +7488 C/T (rs763780)	PCR	Not associated
Mazurek‐Mochol et al. 2021 [46]	Case‐controls	Polish	215/145	46.72	200/160	360	Periodontitis	*IL‐17A*: 197 G/A (rs2275913) *IL‐17F*: +7488 C/T (rs763780)	Real‐Time PCR	Not associated
Kumar et al. 2021 [47]	Case‐controls	Indian	35/25	30–44	30/30	60	Periodontitis	*IL‐17A*: 197 G/A (rs2275913)	RFLP, PCR	Not associated
Hidalgo et al. 2021 [48]	Case‐controls	Brazilian	548/331	49	541/338	879	Periodontitis	*IL‐17A*: 197 G/A (rs2275913), rs3819024 and rs10484879	Real‐Time PCR	Not Associated
Abdelkawy et al. 2019 [49]	Case‐controls	Egyptian	32/28	33.76	40/20	60	Periodontitis	*IL‐17A:* 197 G/A (rs2275913) *IL‐17F:* + 7488 T/C (rs763780)	PCR	Associated Not associated
Vahabi et al. 2017 [50]	Case‐controls	Iranian	102/72	35.9	99/75	174	Periodontitis	*IL‐17A*: 197 G/A (rs2275913)	RFLP, PCR	Not associated
Hatami et al. 2017 [51]	Case‐controls	Iranian	NR	NR	99/75	174	Periodontitis	*IL‐17R*: A‐7383G (rs879576)	RFLP, PCR	Not associated
Linhartova et al. 2016 [52]	Case‐controls	Czech	291/232	49.3	407/154	523	Periodontitis	*IL‐17A*: 197 G/A (rs2275913) *IL‐17F*: +7488 C/T (rs763780)	PCR	Not Associated
Chaudhari et al. 2015 [53]	Case‐controls	Indian	49/56	37.2	70/35	105	Periodontitis	*IL‐17A*: 197 G/A (rs2275913)	PCR	Associated
Zacarias et al. 2015 [54]	Case‐controls	Brazilian	191/122	46.32	140/173	313	Periodontitis	*IL‐17A*: 197 G/A (rs2275913) *IL‐17F*: +7488 C/T (rs763780)	RFLP, PCR	Associated Not associated
Erdemir et al. 2015 [55]	Case‐controls	Turkish	117/120	40.53	147/90	237	Periodontitis	*IL‐17F:* + 7488 C/T (rs763780) *IL‐23R:* R381Q (rs11209026)	PCR	Not associated
Saraiva et al. 2013 [56]	Case‐controls	Brazilian	131/71	14–70	130/72	202	Periodontitis	*IL‐17A*: 197 G/A (rs2275913) *IL‐17F*: +7488 C/T (rs763780) *IL‐23R*: R381Q (rs11209026)	Real‐Time PCR	Associated Not associated for the others
Kadkhodazadeh et al. 2013 [57]	Case‐controls	Iranian	NR	30	113/84	197	Periodontitis Peri‐implantitis	*IL‐17A*: rs10484879	PCR	Associated
Kadkhodazadeh et al. 2013 [58]	Case‐controls	Iranian	NR	38.4	110/83	193	Periodontitis Peri‐implantitis	*IL‐17R*: A‐7383G (rs879576)	PCR	Not associated
Corrêa et al. 2012 [59]	Case‐controls	Brazilian	33/27	43	30/30	60	Periodontitis	*IL‐17A*: 197 G/A (rs2275913) *IL‐17F*: +7488 C/T (rs763780)	RFLP, PCR	Associated Not associated
**Summary of variables included in the study →**		Iran (31.5%) Brazil (21.1%) India (10.5%) Iraq Libya Bulgaria Poland Egypt Czech Republic Turkey (5.2%)	Woman (46.1%) Men (34.3%) NE (19.6%)	14–70 40.43 ± 6.33	2437/1715 Periodontitis: 2315 Peri‐ implantitis: 90 Healthy: 1715	4152	Periodontitis (94.7%) Peri‐implantitis (15.7%)	*IL‐17A*: rs2275913 (68.4%) *IL‐17A*: rs3819024 (5.3%) *IL‐17A*: rs10484879 (5.3%) *IL‐17F*: rs763780 (47.4%) *IL‐17R*: rs879576 (11%) *IL‐23R*: rs11209026 (11%)	PCR (100%) RFLP (36.8%)	Associated (25%) Not associated (75%)

Abbreviations: F^e^, female; M^a^, male; NE, not specific; NR, not reported; PCR, polymerase chain reaction; RFLP, restriction fragment length polymorphism.

### 2.6 Quality Assessment of the Included Papers

S.M.L.M. and C.H.M.B. assessed the quality of the studies independently. For this purpose, the JBI Critical Appraisal Checklist for Case‐Control Studies [60] was used. The JBI checklist evaluates 10 items. Studies were rated at the low score level if they had 1 to 3 of the JBI criteria, moderate 4–7, and high > 8.

## 3 Results

### 3.1 Study Selection

**Figure 1:**
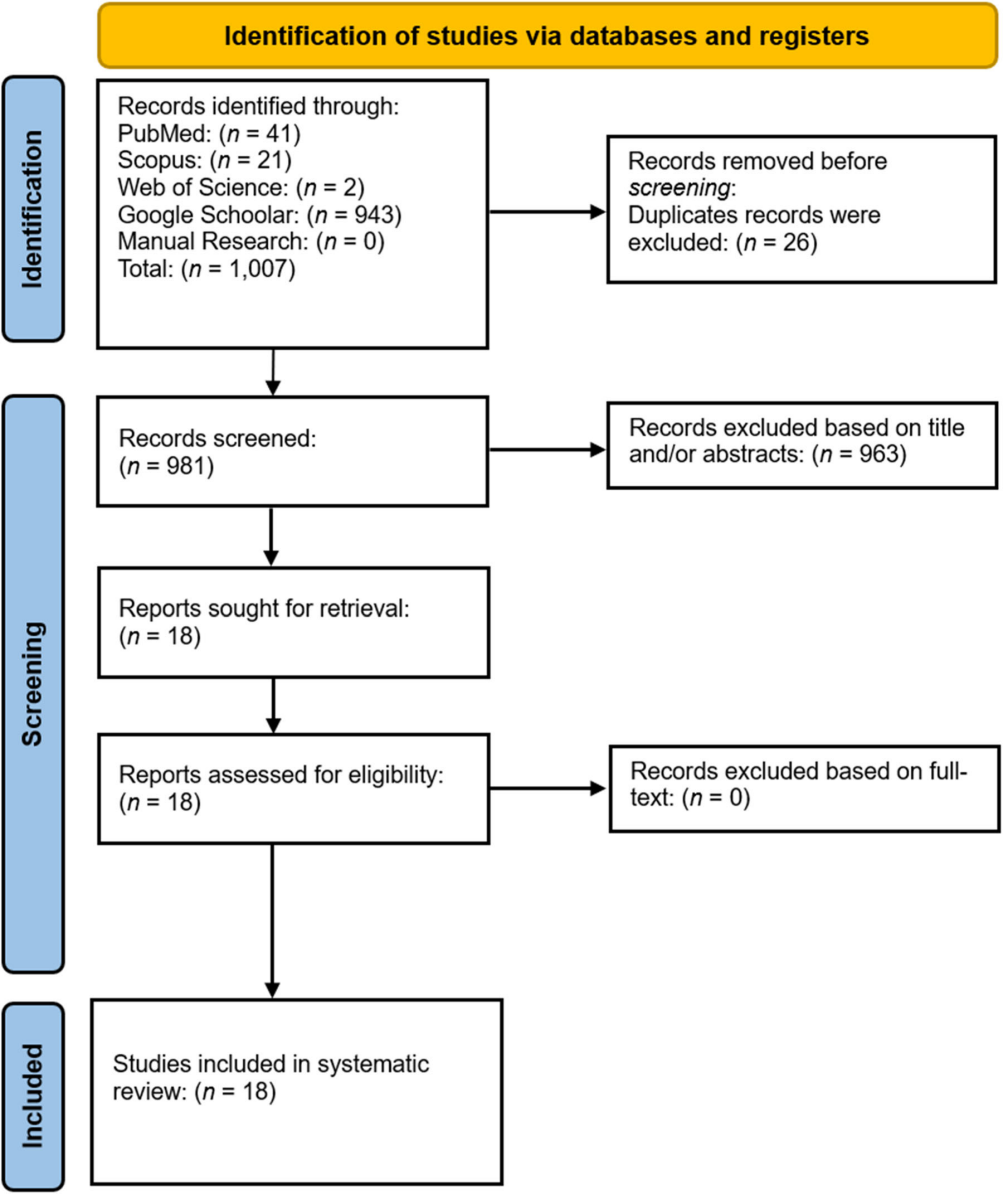
PRISMA workflow used in this systematic review.

The literature search yielded 1007 articles, of which 26 duplicates were excluded. A further 963 articles were excluded after screening of titles and abstracts. A total of 18 remaining articles were retrieved, after which it met the eligibility criteria (Figure 1).

### 3.2 Main Outcomes

Eighteen papers with a case‐control design were those that ultimately met the eligibility criteria. A total of 3904 individuals; 2315 with periodontitis and 90 with peri‐implantitis, and 1589 healthy subjects were studied. The age range of the study population was 14–70 years, with a mean age ± (SD) of 40.43 ± 6.33 years. 46.1% were women, 34.3% were men, and the remainder (16.6%) did not specify gender [51, 57, 58]. Most articles were published after 2013 (17: 94.4%) [42, 43, 44, 45, 46, 47, 48, 49, 50, 51, 52, 53, 54, 55, 56, 57, 58]. Ten different countries were identified where the studies were carried out [42, 43, 44, 45, 46, 47, 48, 49, 50, 51, 52, 53, 54, 55, 56, 57, 58, 59]. Six (33.3%) studies were conducted in Iran [44, 50, 51, 57, 58], four (22.2%) studies were conducted in Brazil [48, 54, 56, 59], and two (11.1%) studies were conducted in India [47, 53]. The rest (33.3%) were conducted in Iraq [42], Libya [43], Bulgaria [45], Poland [46], Egypt [49], Czech Republic [52], and Turkey [55] (Table 2).

This study analyzed a total of twenty‐eight genetic variants corresponding to the *IL‐17A*: rs 2275913 (68.4%), rs 3819024 (5.3%), rs 10484879 (5.3%); *IL‐17F*: rs 763780 (47.4%), *IL‐17R*: rs 879576 (11%) and *IL‐23R*: rs 11209026 (11%) genes in subjects with periodontitis and peri‐implantitis [42, 43, 44, 45, 46, 47, 48, 49, 50, 51, 52, 53, 54, 55, 56, 57, 58, 59]. The most frequent genotyping method was by polymerase chain reaction (100%) [42, 43, 44, 45, 46, 47, 48, 49, 50, 51, 52, 53, 54, 55, 56, 57, 58, 59], followed by the restriction fragment length polymorphism method (38.8%) [44, 47, 50, 51, 54, 59]. Regarding the *IL‐17A* gene, twelve studies evaluated the rs 2275913 upstream variant in 1791 individuals with periodontitis [42, 44, 46, 47, 48, 49, 50, 52, 53, 54, 56, 59] and in 15 individuals with peri‐implantitis [42]. One study evaluated the rs 3819024 upstream variant in 541 individuals with periodontitis [48]. Two studies evaluated the intronic variant rs 10484879 in 616 individuals with periodontitis [48] and 38 with peri‐implantitis [57]. Regarding the *IL‐17F* gene, nine studies evaluated the missense variant rs 763780 in 1184 individuals with periodontitis [43, 45, 46, 49, 52, 54, 55, 56, 59]. Regarding the *IL‐17R* gene, two studies evaluated the missense variant rs 879576 in 172 individuals with periodontitis [51, 58] and 37 with peri‐implantitis [58]. Finally, concerning the *IL‐23R* gene, two studies evaluated the missense variant rs 11209026 in 277 individuals with periodontitis [55, 56] (Table 2).

Six (33.3%) studies found an association between the *IL‐17A*: 197 G/A (rs2275913) genetic variant and peri‐implantitis [53] and periodontitis [49, 54, 56, 59]. One study (5.5%) found an association between the *IL‐17A*: rs10484879 variant and peri‐implantitis and periodontitis [57].

### 3.3 Quality Assessment

**Figure 2:**
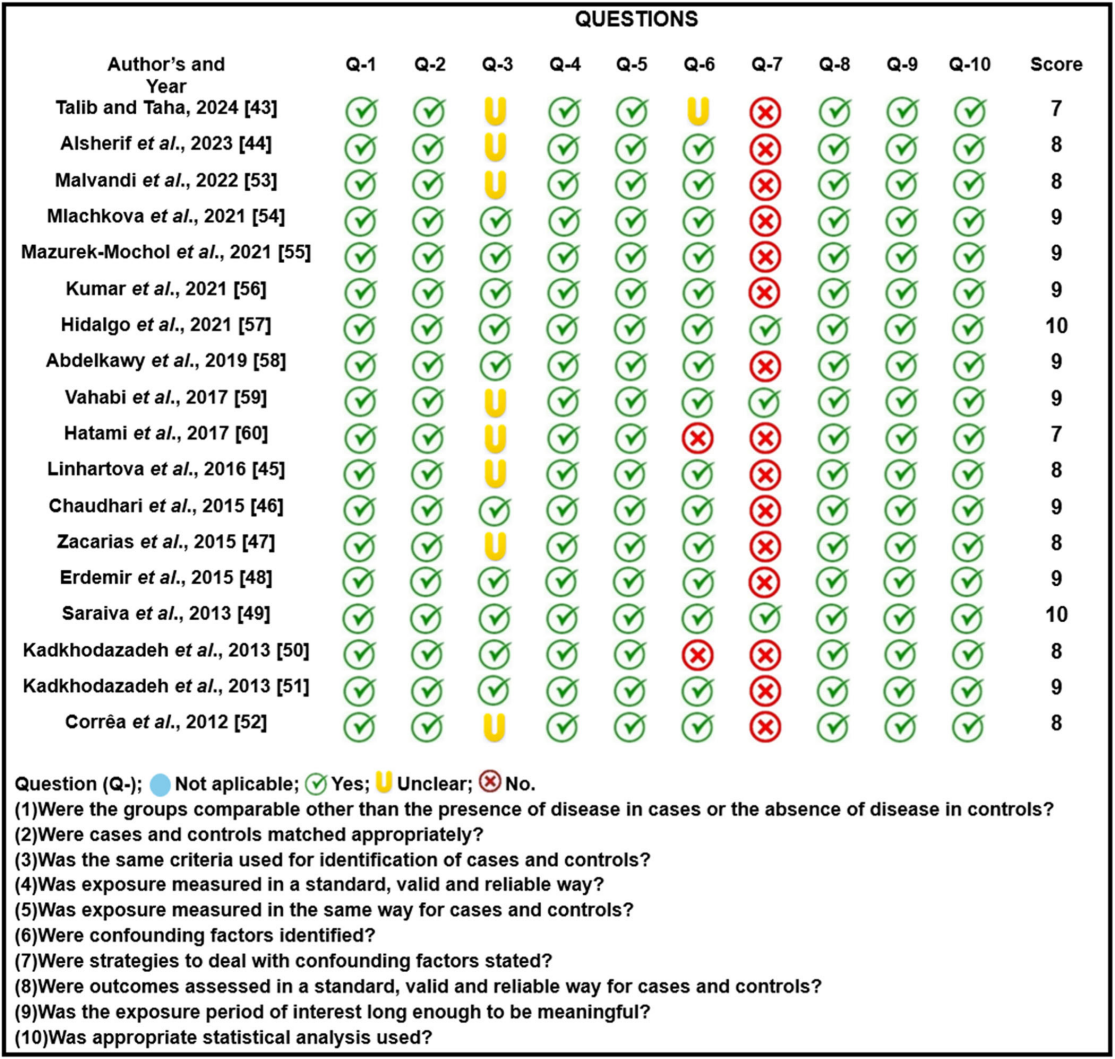
Evaluation of the quality of the articles included in this systematic review according to the JBI items.

Figure 2 shows the results of the JBI Critical Appraisal Checklist. Two articles (11.1%) showed moderate quality [42, 51], and the rest [43, 44, 45, 46, 47, 48, 49, 50, 52, 53, 54, 55, 56, 57, 58, 59] (88.9%) showed high quality. In 44% of the studies [42, 43, 44, 50, 51, 52, 54, 59] it is unclear whether they used specific definitions based on a particular classification system to define the case group. In 16.6% of the studies [42, 51, 57], confounding factors were not identified, and in 83.3% of the studies [42, 43, 44, 45, 46, 47, 49, 52, 53, 54, 55, 57, 58, 59], strategies to address these factors were not specified.

## 4 Discussion

A systematic review was conducted. This study analyzed six genetic variations of IL‐17A, IL‐17F, IL‐17R, and IL‐23R genes in people with periodontitis and peri‐implantitis.

Periodontitis is a disease that damages the supporting tissues of the tooth where bacterial dysbiosis triggers the expression of the IL‐23/IL‐17 axis [22]. Studies reveal that the IL‐23/IL‐17 axis molecules are enhanced in gingival tissue in periodontitis, which correlates with disease development and severity [26].

In periodontal inflammation, the immune system, through dendritic cells and macrophages, produces IL‐23, which by binding to its receptor IL‐23R on Th17 cells stimulates the production of IL17 [61, 62]. IL‐17 mainly stimulates fibroblasts by binding to its receptor, which promotes the expression of more pro‐inflammatory cytokines and one of them is RANKL, which activates osteoclasts and thus promotes bone erosion [61, 62, 63, 64], a frequent and common characteristic in both periodontitis and peri‐implantitis. IL‐23 induces the Th17 pathway in periodontal disease, contributing to the inflammatory response and tissue destruction [65].

In the present review, 15 studies were in patients with periodontitis [43, 44, 46, 47, 48, 49, 50, 51, 52, 53, 54, 55, 56, 57, 58, 59], two studies evaluated polymorphisms in both conditions [57, 58], and one study only evaluated patients with peri‐implantitis [42].

Regarding IL‐23R, two articles in this review evaluated the polymorphism (rs11209026) for which they did not observe an association with the risk or aggravation of periodontitis [55, 56]. It is important to note that exon 9 of the IL‐23R receptor gene encodes a polymorphism where the presence of the A allele, instead of the G allele, results in an amino acid change from arginine to glutamine at residue 381 (R381Q) [66]. As a result, substituting glutamine (Gln) for arginine (Arg) alters IL‐23 signaling and responses. The substitution of arginine (Arg) for glutamine (Gln) takes place in the cytoplasmic portion of the IL‐23R, near the initial site of tyrosine phosphorylation, between the JAK2 binding site and the transmembrane domain [66, 67].

Considering the role of the IL‐23 gene in Th17 cells, some modifications in its gene can impact its transcription and translation, which can generate an inefficient receptor.

Various studies have evaluated this polymorphism in some autoimmune diseases such as Crohn's disease, ankylosing spondylitis, and psoriasis. The IL‐23‐R381Q variant is considered to provide protection against autoimmunity and inflammation [67, 68, 69, 70, 71]

It has been reported that the G to A variant of the minor allele introduces an amino acid change (Arg381Gln) in the intracellular domain of IL‐23R, near the JAK2 kinase binding site. T cells that present the minor allele have IL‐23‐dependent phosphorylation of STAT3, therefore these cells express IL‐17 less. On the other hand, individuals with this variant have a lower quantity of Th17 cells [67].

On the other hand, soluble variants of the IL‐23R receptor have also been found. Soluble IL‐23R can be generated by alternative splicing [72] or proteolytic cleavage [73]. Once the IL‐23R receptor is soluble, it can inhibit IL‐23 by forming a complex before the IL‐23 binds to a receptor on the TH17 membrane [74, 75].

Regarding IL‐17A, three polymorphisms were evaluated in the studies of this systematic review. The rs2275913 polymorphism was the most studied. Six articles mentioned an association with periodontitis [42, 49, 53, 54, 56, 59], and six articles reported no association [44, 45, 47, 50, 51, 76].

In periodontitis, Hidalgo et al evaluated the rs2275913, rs3819024, and rs 10484879 polymorphisms of IL‐17A and observed that there is no association with developing periodontitis in the presence or absence of type 2 diabetes mellitus [48]. However, when the total number of participants was stratified by smoking behavior, smokers and former smokers were shown to have the GG genotype of the SNP rs3819024 significantly more frequently than healthy people than periodontitis and Type 2 Diabetes mellitus patients [55].

Regarding the ra10484879 polymorphism, Kadkhodazadeh reports a higher CC genotype frequency than CA and AA in patients with periodontitis, peri‐implantitis, and controls. Furthermore, it was observed that the AA genotype was absent in the chronic periodontitis and peri‐implantitis groups, while it was detected in the control group [57].

On the other hand, it has been reported that in the Egyptian population, the A allele of the rs2275913 polymorphism may be a risk factor for periodontal diseases and, therefore, this polymorphism could be a risk predictor for patients with stage II and III periodontitis [49]. These results agree with those reported by Correa et al, Chaudhari et al, and Zacarias et al, in addition to the fact that patients with this polymorphism show elevated levels of IL‐17A [53, 54, 59]. On the contrary, Linhartova also evaluated the rs2275913 polymorphism in patients with periodontitis, type 2 diabetes mellitus, and control subjects, and did not observe an association with patients with periodontitis but with patients with type 2 diabetes mellitus [52]. Contrary to the above, Saravia et al reported that the rs2275913 genotype GG polymorphism is more frequent in patients with chronic and aggressive periodontitis [56].

It is worth mentioning that various studies have reported the association of the rs2275913 polymorphism with different pathologies such as colorectal cancer and rheumatoid arthritis among other diseases [77, 78]. Likewise, a risk association has been observed in the airways such as asthma, severity due to COVID‐19, and resistance to antituberculosis drugs. Furthermore, the presence of this polymorphism in arthritis and patients resistant to antituberculosis drugs generates an increase in serum levels of IL‐17A [79, 80, 81], as observed by Correa in serum and gingival tissue [59]. In addition to this, it has been observed that the serum levels of IL‐17A in chronic periodontitis patients with allele A are greater than in patients with allele G [53].

It is important to note that the findings between the rs2275913 polymorphism and periodontal disease may still be considered inconclusive. This is because, of the 12 studies that evaluated this polymorphism, half found an association and the other half did not. It is worth mentioning that the articles that do not report an association with periodontal disease are studies with a larger sample size. The total number of participants in studies without an association between periodontitis and the rs2275913 polymorphism was 2168 and only 785 participants did show an association. Therefore, we could infer that an association of this polymorphism with periodontitis is less likely. Regarding the studies with the largest sample size, two were carried out in Iran [44, 50], and the rest in Polish [46], Indian [47], Brazil [48, 54, 56] and Czech [52]. If the studies were in patients from the same country or geographical area, we could propose that the lack of association of this polymorphism is merely for a genetic reason or association with a race; however, the participating population with an association between periodontitis and the polymorphism of periodontitis is quite heterogeneous *IL‐17A* rs2275913.

The effect of interleukin‐17 gene polymorphisms on periodontitis has sparked considerable research. Research suggests that IL‐17 gene polymorphisms, such as the A‐197G polymorphism, are associated with increased IL‐17 levels in chronic periodontitis patients [82]. This overexpression of IL‐17 is thought to have a role in the development of chronic periodontitis by increasing neutrophil recruitment, activating the release of inflammatory mediators, and aiding alveolar bone resorption [59]. It may affect the production of IL‐17, thereby altering the inflammatory responses found in periodontitis [46].

IL‐17A can form a heterodimer with IL‐17F and signal at the IL‐17RA or IL‐17RC receptor, even these cytokines can signal to these receptors in a homodimeric manner [16, 18]. Therefore, not only IL‐17A may have some association with periodontitis or peri‐implantitis. Therefore, several authors evaluated the rs763780 polymorphism of IL‐17F [43, 45, 46, 49, 52, 54, 59, 83, 84] of which 5 of 9 studies did find an association with periodontitis [48, 49, 59, 83, 84].

In patients with periodontitis as well as peri‐implantitis no association with this polymorphism was observed [51, 57]. Given that the receptor is required for IL‐17 signaling, regardless of whether the cytokine is overexpressed or not, it indicates that this polymorphism does not affect the receptor in any compromised region. The extracellular or cytoplasmic area that might hinder its interaction with the cytokines IL‐17A/IL‐17F or the related signaling pathway would not be used. In addition, it is known that there is an isoform of soluble IL‐17RA, and this receptor, like soluble IL‐23R, may bind to IL‐17 and block it [85, 86].

## 5 Conclusion

Six polymorphisms were evaluated, the most studied was rs 2275913 of the cytokine IL‐17A in patients with periodontitis or peri‐implantitis. Only 50% of the articles found an association, however, the sample size of these studies is smaller compared to studies that did not observe an association between the rs 2275913 polymorphism with periodontitis or peri‐implantitis. Therefore, it is considered that another factors may determine the association with these periodontal conditions, such as the degree or stage of the disease, the presence of any systemic disease, and even the ethnic group studied.

## Author Contributions

Conceptualization: Ruth Rodríguez‐Montaño, Artak Heboyan, and Mario Alberto Alarcón‐Sánchez. Methodology: Mario Alberto Alarcón‐Sánchez. Software: Mario Alberto Alarcón‐Sánchez. Validation: Ruth Rodríguez‐Montaño, Mario Alberto Alarcón‐Sánchez, and Artak Heboyan. Formal analysis: Ruth Rodríguez‐Montaño, Mario Alberto Alarcón‐Sánchez, Sarah Monserrat Lomelí‐Martínez, and Cristina Hermila Martínez‐Bugarin. Investigation: Ruth Rodríguez‐Montaño and Mario Alberto Alarcón‐Sánchez. Resources: Ruth Rodríguez‐Montaño, and Mario Alberto Alarcón‐Sánchez. Data curation: Mario Alberto Alarcón‐Sánchez. Writing–original draft preparation: Ruth Rodríguez‐Montaño, Mario Alberto Alarcón‐Sánchez, Sarah Monserrat Lomelí‐Martínez and Cristina Hermila Martínez‐Bugarin. Writing–review and editing: Ruth Rodríguez‐Montaño, Mario Alberto Alarcón‐Sánchez, and Artak Heboyan. Visualization: Ruth Rodríguez‐Montaño, Mario Alberto Alarcón‐Sánchez, Sarah Monserrat Lomelí‐Martínez, Cristina Hermila Martínez‐Bugarin, and Artak Heboyan. Supervision: Ruth Rodríguez‐Montaño, Mario Alberto Alarcón‐Sánchez, and Artak Heboyan. Project administration: Ruth Rodríguez‐Montaño, Mario Alberto Alarcón‐Sánchez, and Artak Heboyan. All authors have read and agreed to the published version of the manuscript.

## Ethics Statement

The authors have nothing to report.

## Consent

The authors have nothing to report.

## Conflicts of Interest

The authors declare no conflicts of interest.

## Data Availability

The data supporting this study's findings are available from the corresponding author upon reasonable request.
